# Medical History from the Earliest Times

**Published:** 1892-09-17

**Authors:** E. T. Withington


### MEDICAL HISTORY FROM THE EARLIEST TIMES.

By E. T. WITHINGTON, M.A., M.B. Oxon.

### XXIV.—THE INFLUENCE OF CHRISTIANITY ON MEDICINE.

IT must be admitted that the triumph of our holy religion—though by no means so responsible for the decline of medicine as is sometimes asserted—nevertheless tended to hasten, rather than to hinder that downfall; but by means of one great institution it has, in the long run, done far more to further the healing art than it ever did to injure it.

We have found traces of establishments for the sick in ancient India; the Greeks possessed public "Iatreia," which can scarcely be distinguished from rate-supported infirmaries; and Prescott tells us that the Spaniards met with "hospitals" among the Aztecs of Mexico. But there is, I believe, no certain evidence of any medical institution supported by the voluntary contributions of large numbers of people, or by private munificence, till we come to Christian days. The Primitive Church, as we learn from the "Acts," cared for its poor from the earliest time, but we find no definite notice of any special buildings for the reception of the sick till the close of the fourth century. St. Jerome, writing about A.D. 400, says that Fabiola, a noble Roman lady and his own friend, was the first to build a hospital. He tells us that she had divorced her first husband and married again, but, being left a widow, publicly repented of her sin, sold all her possessions, and established a hospital for the sick in which she herself laboured. "How often did she carry the sick on her own shoulders! How often did she wash the putrid matter from wounds another could not have borne to look upon! With her own hands she prepared their food, and moistened with water the parched lips of the dying."

But the grandest effort of early Christian philanthropy was the great "Ptochotrophion," or Almshouse of St. Basil, Bishop of the Cappadocian Cæsarea (370-379), though unfortunately we know nothing of its medical arrangements, except that it had special physicians, nor in how far the bishop was supported by the contributions of his flock. The humility of St. Basil has left us only the letters in which he defends his expenditure, and pleads for exemption from taxes, and his silence is but partially atoned for by the florid eloquence of St. Gregory Nazianzen. That enthusiastic orator bids his hearers go forth from the gate of Cæsarea and they will find another city, before which the seven wonders of the pagan world sink into insignificance. Here the saintly bishop had gathered together the poor, the fallen, the sick, and the afflicted from all parts of Christendom, nor did he hesitate to kiss and embrace the most loathsome of lepers, not out of ostentation, but in token that they also were his brothers in Christ. At a yet earlier period we hear of communities both of men, and women, "parabolani" and deaconesses, who sought out the sick and tended them in their own homes, and who in times of pestilence attracted even the notice of the heathen by their courage and devotion. But the parabolani unfortunately too often degenerated into body guards of turbulent bishops, whom they assisted in asserting or refuting theological doctrines by the physical arguments of their swords and cudgels.

And this brings us to a less attractive side of our subject, the consideration of the unfavourable influences which the new religion had upon medicine. These acted in three principal ways; first, by helping to restore the primitive theories of disease; secondly, when Christianity had fully triumphed, by imposing restrictions on freethought and investigation; and, thirdly, by giving rise to religious controversies so widespread and vehement that they sometimes seemed to absorb all the intellectual energies of the age. Of these the first was, at the time now treated of, much the most prominent, but it must always be remembered that the evil work had to a great extent already been accomplished by the Gnostics and Neoplatonists. Far be it, too, that we should in any way exaggerate this influence, or bring forward chance references to demons, incubi, and the like, as examples of the early faith of Christendom. Let us rather confine ourselves to the writings of one of the most cultured of the Eastern fathers, the great Bishop of Cæsarea himself.

St. Basil was of a weakly constitution, much liable to illness, and had therefore paid great attention to medicine, which he declares is the noblest of all worldly professions. He had in all probability read the famous treatise on epilepsy, in which the Hippocratic writer, 800 years before St. Basil's day, so clearly proclaimed the natural origin of all disease, and thereby assisted in producing a revolution in medicine. Yet the bishop begins by flatly contradicting him. Not all diseases, he says, are produced by nature, or even by our own vices and bad habits; some are sent directly from God Himself as trials of our faith or punishments for some forgotten sin (1 Cor. xi. 30), and whenever we are conscious of this we should by no means go to a physician, but bear patiently the chastening of the Lord till He sees fit to remove it (Micah vii. 9). And more than this; some diseases may, by the permission of God, be caused by Satan (Job ii. 6, 7), and here also our chief object should be to emulate the patience of the patriarch. The good bishop, however, did not let his theories interfere with his provision of medical aid for the poor, and in the East the Hippocratic tradition was still strong enough to neutralise the worst effects of these doctrines. But when we come to consider the corresponding period in Western Europe, we shall find this theologic pathology still further developed, and joined to a theologic therapeusis and a belief in the sole efficacy of prayer and the relics of the saints, which, had it been carried to its logical conclusion, would have made medicine not only a useless but an impious profession.

Among the more prominent forms of pagan religion the worship of the medical divinities seems to have lingered longest; nor did the opponents of Christianity hesitate to compare the cures wrought in the temples of Æsculapius and Serapis with the miracles of saints and martyrs, and even with those recorded by the Evangelists. It was natural, therefore, that the Church, as soon as she obtained control of the secular arm, should proceed to attack these presumptuous rivals; but it was not until a century after the conversion of Constantine that the practice of "incubation" in heathen temples was finally suppressed by the decrees of the emperors and the violence of the Christians. The latter, however, soon began to imitate the practices they had condemned. Models of healed limbs, &c., formerly suspended in temples, were now dedicated in churches, and the dream oracles, sought of old at the shrines of Æsculapius and Serapis, were invoked in the convents of mediæval Italy and the churches of modern Greece. The two following "clinical histories" may help to show how closely pagan and Christian medico-religious beliefs corresponded. The first is from one of many similar inscriptions found in the temple of Æsculapius at Epidaurus, and dates from the fourth century B.C. "Gorgias of Heraclea Empyema. This man, being wounded in the lung in a certain battle, carried the weapon in his body for eighteen months, and discharged sufficient pus to fill sixty-seven porringers. He came to the temple, and when asleep he saw a vision: the god seemed to extract the spearpoint from his lung and to place it in his hand, and in the morning he went away whole, taking the weapon with him." The second is from the life of Meinwerc, Bishop of Paderborn, in the eleventh century. In the year 1014 St. Henry, then German King and Emperor-elect, being grievously tormented by stone, came to the monastery of Monte Cassino and prayed earnestly that he might be healed. While asleep he saw a vision: St. Benedict, founder of the monastery, appeared to him and told him his prayers had been heard in heaven. Then, with a surgeon's knife (ferro medicinali), he opened the part where the stone was, took it out, and placed it in the king's hand, and, the wound being miraculously healed, he disappeared. The king awoke, found the stone in his hand, and calling the bishops and princes, told them what great things God had done for him. Then, having given royal gifts to the monastery, he departed rejoicing, and came to Rome, where he told the story to Pope Benedict VIII., who rejoiced with him, and crowned him Cæsar and Emperor February 14th, 1014.

